# Unsupervised and explainable machine learning solutions for last-minute cancelations in pediatric ambulatory and day surgeries in Italy

**DOI:** 10.3389/frai.2026.1743396

**Published:** 2026-04-13

**Authors:** Marco Cascella, Cosimo Guerra, Rosario De Feo, Riccardo Tarquini, Elisa Francia, Ilaria Mascilini, Cecilia M. Pizzo, Angela Galeotti, Martina Caputo, Roberto Pedone, Franco Marinangeli, Francesco Sabbatino, Giuliano Marchetti, Corrado Cecchetti, Rajeev S. Iyer, Alessandro Vittori

**Affiliations:** 1 Unit of Anesthesia and Pain Medicine, Department of Medicine, Surgery and Dentistry “Scuola Medica Salernitana”, University of Salerno, Baronissi, Salerno, Italy; 2 Department of Medicine, Surgery, and Dentistry “Scuola Medica Salernitana”, University of Salerno, Baronissi, Salerno, Italy; 3 Department of Anesthesia, Critical Care, and Pain Medicine, ARCO, Ospedale Pediatrico Bambino Gesù IRCCS, Rome, Italy; 4 Dentistry Unit, Ospedale Pediatrico Bambino Gesù IRCCS, Rome, Italy; 5 Department of Psychology, University of Campania Luigi Vanvitelli, Caserta, Italy; 6 Department of Anesthesiology, Intensive Care, and Pain Treatment, University of L'Aquila, L’Aquila, Italy; 7 Oncology Unit, Department of Medicine, Surgery, and Dentistry, University of Salerno, Baronissi, Salerno, Italy; 8 Surgery Unit, Bios Medical Center, Rome, Italy; 9 Department of Anesthesiology and Critical Care Medicine, Children’s Hospital of Philadelphia, University of Pennsylvania, Perelman School of Medicine, Philadelphia, PA, United States

**Keywords:** ambulatory anesthesia, artificial intelligence, canceled procedure, day surgery, explainable AI, machine learning, pediatric anesthesia, pediatric surgery

## Abstract

**Background:**

Canceled procedures in pediatric day-case surgery are a serious issue that can disrupt hospital workflow, resource utilization, and patient care. To better analyze this and explore strategies to mitigate such costly inefficiencies, we employed unsupervised machine learning (ML) techniques to analyze data from children undergoing day-case surgery at the Ospedale Pediatrico Bambino Gesù IRCCS between January 2020 and March 2022.

**Methods:**

We analyzed a dataset including 4,417 operated and 183 non-operated patients. The following variables were considered: age, hospitalization type, surgical specialty, procedure type, and reasons for cancelation. Dimensionality reduction was performed using Factor Analysis of Mixed Data (FAMD). Cluster analysis was conducted using the k-means algorithm, while a Random Forest classifier was employed to assess feature importance and enhance model interpretability.

**Results:**

The overall cancelation rate was 3.84%. Among operated patients, the median duration of surgery was 22 min (IQR: 12–32). For non-operated patients, the median waiting time from first consultation to the scheduled surgical procedure was 105 days (IQR: 58–194). K-means clustering (*k* = 3) identified three distinct patient groups, supported by robust clustering metrics (silhouette score: 0.696; Davies–Bouldin index: 0.447; Calinski–Harabasz score: 10,119.546). The Hopkins statistic (0.003) confirmed a strong clustering tendency in the dataset. Cluster 0 (*n* = 2,010) was mainly characterized by plastic and maxillofacial procedures (*n* = 765), andrological procedures (*n* = 533), and pediatric urology surgeries (*n* = 194), largely performed in an ambulatory setting (*n* = 1,779). Cluster 1 (*n* = 1,773) predominantly included andrological procedures (*n* = 809) and showed the longest median intervention duration (27.01 min). Significant inter-cluster differences were observed for age (Kruskal–Wallis test, *p* < 0.001 for clusters 0–1 and 1–2), surgical intervention rates (highest in Cluster 2: 97.92%; *χ*^2^ = 10.11, *p* = 0.0063), and procedure duration (all pairwise Dunn tests *p* < 0.001). Random Forest analysis identified hospitalization type (feature importance: 0.47) and procedure type (feature importance: 0.45) as the most influential variables contributing to cluster differentiation.

**Conclusion:**

The ML analysis may suggest targeted strategies for optimizing scheduling and resource allocation, ultimately improving patient care and operational efficiency.

## 1 Introduction

The effects of day surgery and ambulatory procedure cancelations on the day of the procedure can have a significant impact on patients, their families, and healthcare systems (Solak et al., 2019; Ezike et al., 2011). The last-minute cancelation, defined as cancelations the same day of procedures (Vittori et al., 2025), has an even more dire impact on both patients and their families, as well as on the healthcare system. Last-minute cancelations can cause anxiety, emotional distress, and frustration for both patients and families (Olson and Dhakal, 2015). Moreover, families face logistical challenges, including rearranging work schedules, organizing transportation, and accommodating additional caregiving responsibilities (Koushan et al., 2021). More importantly, delays in treatment may exacerbate the underlying medical condition, potentially leading to worse health outcomes (Wolfler et al., 2020). Furthermore, cancelations can impair the scheduling process, delaying care for other patients (Koh et al., 2021). Additionally, rescheduling procedures may increase the risk of complications due to prolonged waiting times, as well as the potential for medical conditions to worsen while awaiting surgery (Al Talalwah and McIltrot, 2019).

In this complex scenario, a clustering analysis can be instrumental in identifying priority intervention areas and ultimately preventing procedure cancelations (Aguilar and Barbosa, 2023). A cluster analysis is a statistical technique used to group data points into clusters based on their similarities, helping to identify hidden patterns or structures within a dataset. In machine learning (ML), clustering is an unsupervised learning method as it does not require labeled data. ML algorithms, such as k-means and hierarchical clustering, automatically analyze large datasets to detect natural groupings (Bellini et al., 2022). The role of ML in cluster analysis is to enhance efficiency, accuracy, and scalability, making it easier to process complex datasets and extract meaningful insights for strategic decision-making (Pollard and Olson, 1999; Ferschl et al., 2005; Compère et al., 2017).

To address the issue of surgery cancelation, clinicians can adopt this unsupervised ML strategy to segment patients with similar characteristics, such as medical history, risk factors, and urgency levels. Therefore, healthcare providers could proactively address the issues of cancelation before they arise by analyzing clusters of patients who have a history of cancelations. Hospitals can pinpoint recurring reasons, such as incomplete preoperative assessments, last-minute infections, or administrative issues, and implement targeted preventive measures (Ezike et al., 2011). Furthermore, understanding patient clusters enables hospitals to allocate resources effectively, ensuring that high-risk groups receive additional monitoring or early interventions to reduce the rate of cancelations (Pohlman et al., 2012). Risk clustering can also identify subgroups of patients who may require enhanced preoperative preparation, such as specific medical evaluations, parental education, or social support. Ultimately, clustering analysis provides data-driven insights that enable hospitals to implement targeted interventions, reduce inefficiencies, and enhance the workflow of pediatric surgical care.

With this background, this study aimed to analyze the factors that may lead to last-minute cancelations in pediatric day surgery and ambulatory surgery by applying cluster analysis with an explainable approach.

## 2 Methods

This study included all children who underwent day-case and ambulatory procedures at the *Ospedale Pediatrico Bambino Gesù IRCCS* in Rome, Italy, between January 2020 and March 2022. All procedures adhered to the ethical standards of the Declaration of Helsinki and its subsequent amendments. Ethics Committee of the Ospedale Pediatrico Bambino Gesù IRCCS (Ethics Committee protocol n° 888, Chairman Professor Alessandro Nanni Costa) approved this study on 04 October 2023. Ethics Committee of Ospedale Pediatrico Bambino Gesù IRCCS waived the requirement of informed consent, given the retrospective nature of the study and the complete anonymization of the data. The study data are a secondary analysis of our previous study on the risk factors of last-minute cancelations in pediatric ambulatory and day surgeries in Italy (Vittori et al., 2025).

### 2.1 Data preprocessing

The original dataset (Vittori and Cascella, 2024) was divided into two subsets: patients who underwent surgery (operated patient dataset) and those who had their procedure canceled (non-operated patient dataset).

- Operated patients’ dataset: Included 4,417 patients and 18 variables, including the date of surgery, patient age, operating block, start and end times of the procedure, duration, surgical sector, and type of procedure.
- Non-operated patients’ dataset: Included 183 patients with similar variables and included data capture for “Reason for Cancelation.”

The analysis focused on the following variables: age, procedure duration (for operated patients), surgical team, procedure code, hospitalization type, and reason for cancelation (for non-operated patients).

We merged the datasets and created new categorical variables. We created “setcat” for categorizing surgical teams into 16 categories and “codcat” for categorizing procedure codes into 101 classes. “Performed” was used as a binary variable to indicate whether the surgery was performed (1) or not (0); “Hospitalization Type” was categorized into a categorical variable called “HospitalizationRegime” into three classes: ambulatory surgeries (0), single-day hospital access (1), and multiple-day hospital access (2).

In Italy, ambulatory surgery is defined as a surgical or diagnostic procedure that does not require hospitalization (Vittori et al., 2025; ASAHQ, 2024). Day Surgery is defined as a surgical or diagnostic procedure that requires daytime hospitalization (Vittori et al., 2025; AGENAS, 2024). In cases requiring further diagnostic investigations, such as comorbidities, patients require multiple-day hospital access.

### 2.2 Statistical analysis, including clustering analysis

To reduce dimensionality and facilitate clustering, the Factor Analysis of Mixed Data (FAMD) technique was applied (RPubs, 2025). This approach is particularly suitable for datasets with both categorical and continuous variables (Pagès, 2014). Before proceeding with clustering, our statistician performed a cluster tendency assessment to determine whether the data structure was suitable for meaningful clustering. The Hopkins score was calculated, and scores close to zero indicated a high tendency for clustering. We chose the k-means algorithm for clustering due to its efficiency and suitability for large datasets. To determine the optimal number of clusters (*k*), multiple metrics were evaluated, including the silhouette score, Davies-Bouldin index, and Calinski-Harabasz score (Davies and Bouldin, 1979; Caliński and Harabasz, 1974). To validate the differences among clusters, statistical tests were performed on numerical and categorical variables. Specifically, we applied the Kruskal-Wallis test to assess significant differences in age across clusters, and followed up with post-hoc Dunn tests using the Bonferroni correction (Kruskal and Wallis, 1952). We evaluated the differences in categorical variables with Chi-square tests.

As a cluster explainability approach, a Random Forest classifier was trained on clusters’ membership labels; feature importances were extracted to determine which features had mostly driven clusters’ assignation.

The Clustering analysis was conducted using Python (v3.12.7) with the following packages: *seaborn*, *matplotlib* for visualization; *scipy* for Statistical Analysis; *scikit-learn*, *pyclustertend* (Hopkins score calculation for cluster tendency) for clustering; and *prince* for FAMD.

## 3 Results

**Figure 1:**
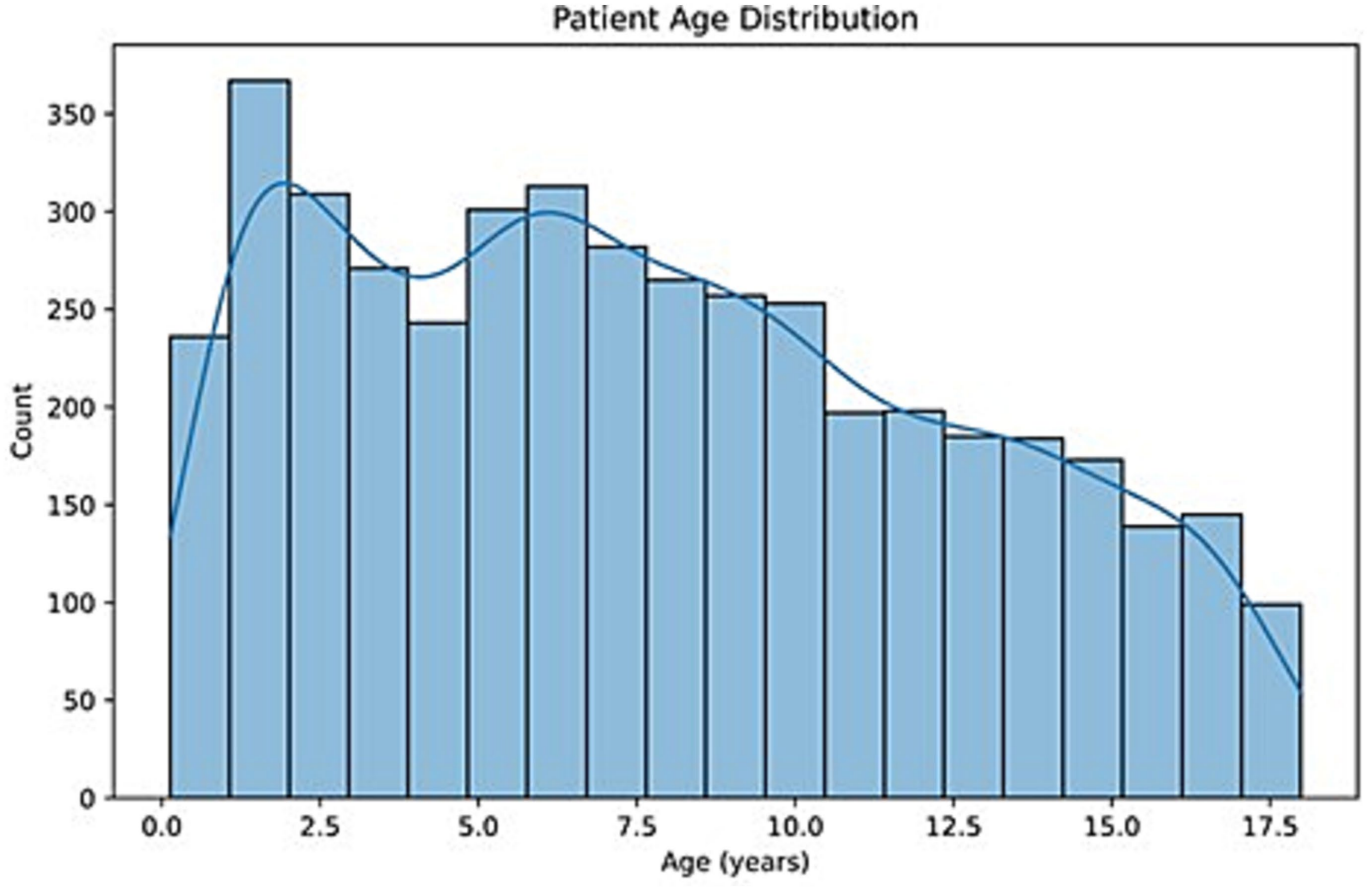
Distribution of patient ages.

The obtained dataset included findings from 4,600 cases. The rate of case cancelations was 3.84%. The age of the patients showed substantial variability, ranging from 1 month to 18 years (0.13–17.85 years). The median age was 6.54 years, with an interquartile range (IQR) of 2.67–10.57 years (Figure 1).

**Figure 2:**
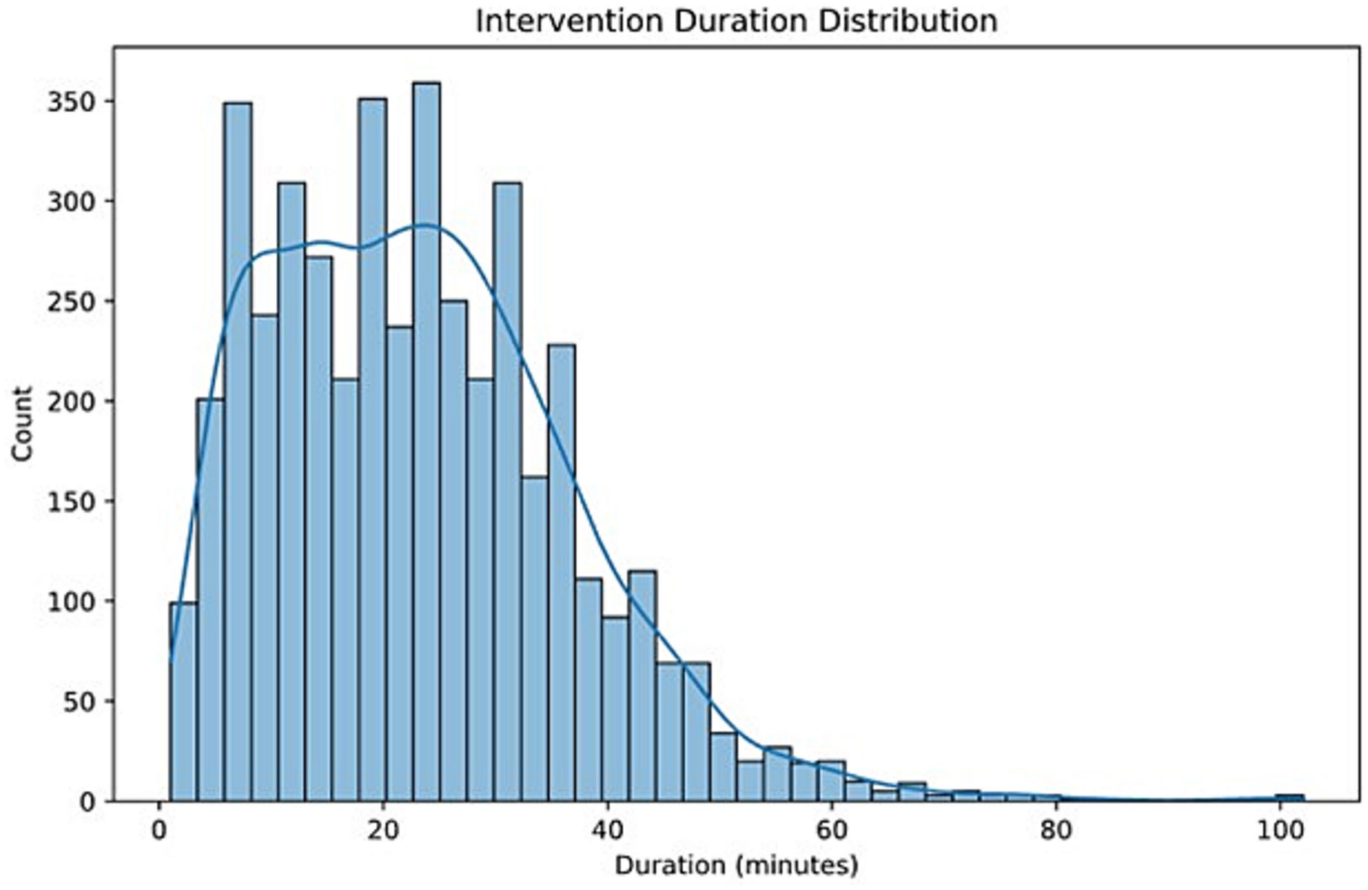
Intervention duration calculated in minutes.

Among the patients operated on, the median intervention duration was 22.01 min (IQR 20.0, 12.01–32.01) (Figure 2).

When examining the canceled procedures, we calculated the waiting time to surgery as the time interval between the first consultation date and the procedure date, expressed in days. Approximately 6% of patients (10/183) did not have a recorded date of the first surgical consultation, and one patient did not have a valid date; hence, they were excluded from this calculation.

**Figure 3:**
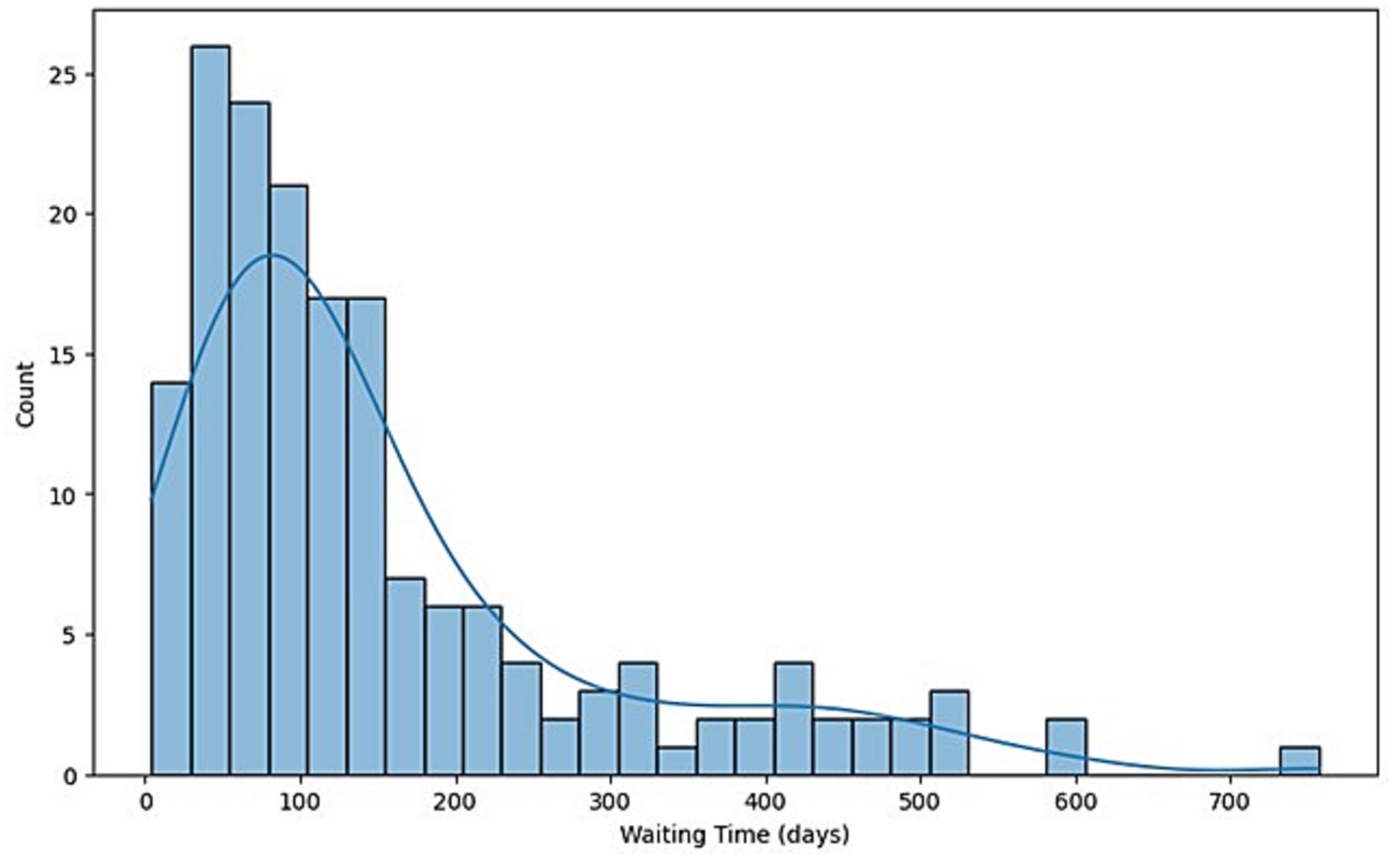
Distribution of median waiting times in days among non-operated patients.

The median waiting time from the first consultation to surgery was 105 days (IQR: 58–194 days), with the shortest interval being 4 days and the longest 757 days (Figure 3).

**Figure 4:**
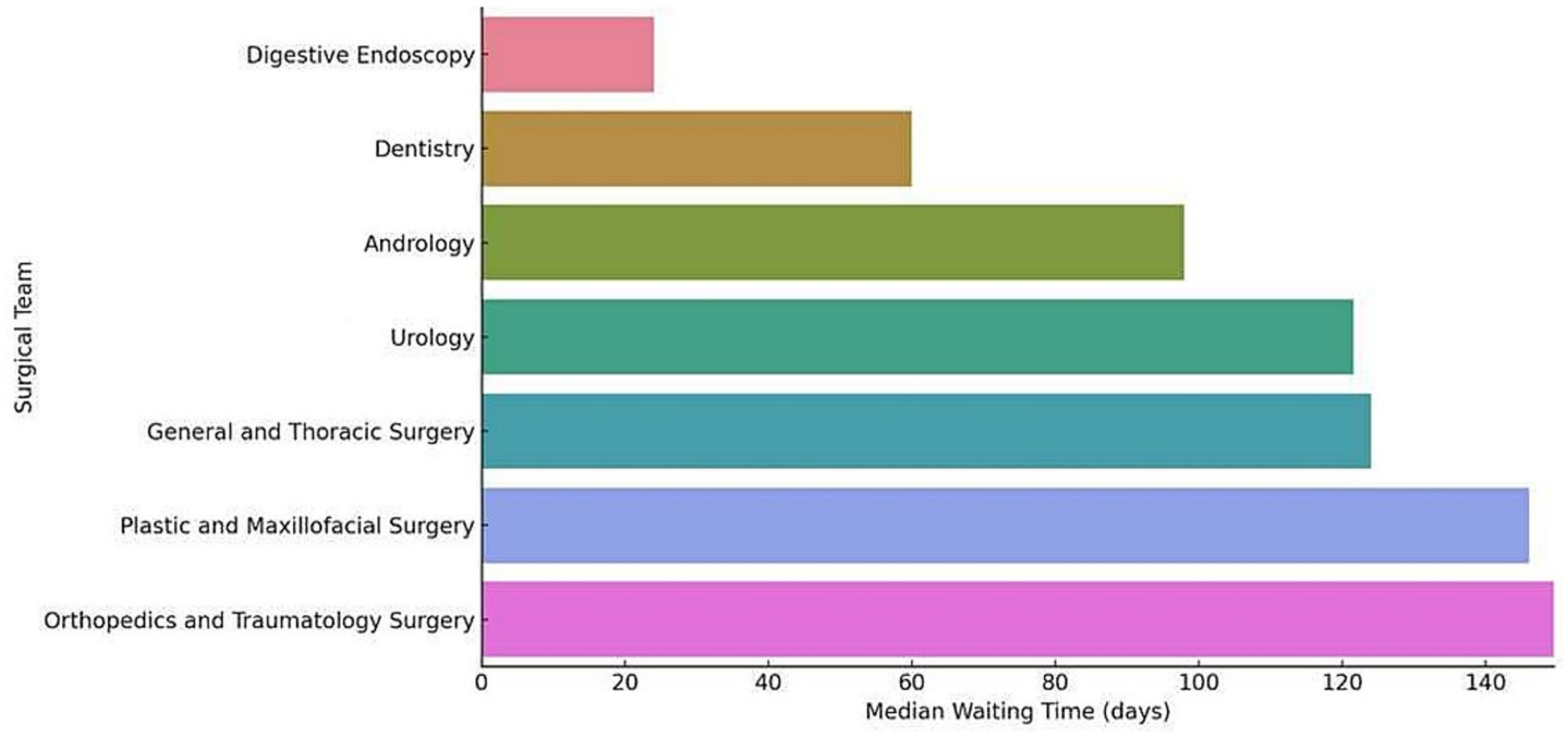
Median waiting time (days) by surgical team.

In these same patients, analysis of waiting times by surgical team revealed considerable variability between teams, reflecting possible differences in priorities, procedural complexities, and resource availability. For instance, the median waiting time for the Digestive Endoscopy team was 24 days, while the Orthopedic team reported a median of 149.5 days. Other teams, such as Dentistry, Andrology, and Urology, exhibited median waiting times of 60, 98, and 121.5 days, respectively. In contrast, the General and Thoracic Surgery team and the Plastic and Maxillofacial Surgery team reported median waiting times of 124 and 146 days, respectively (Figure 4).

**Figure 5:**
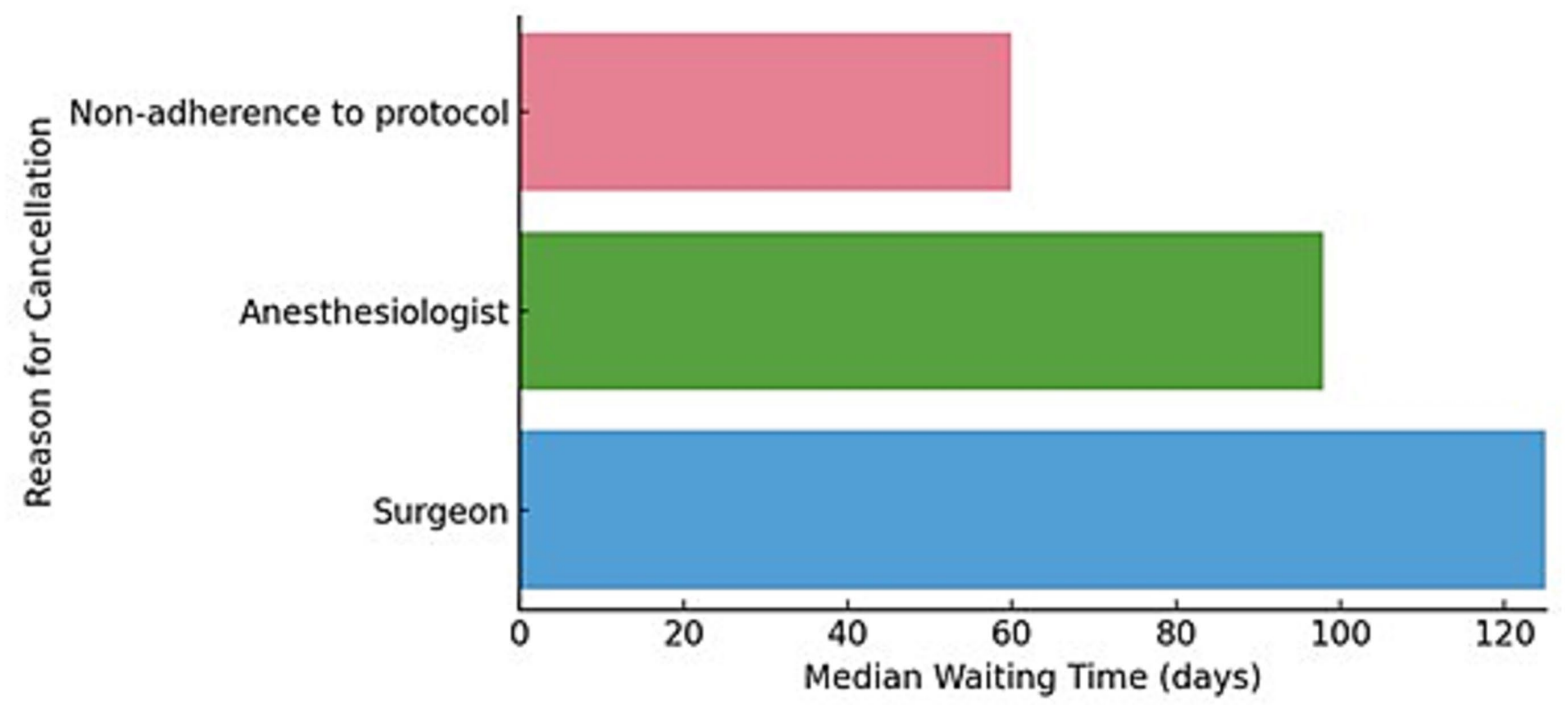
Median waiting time by reason for cancelation.

Most procedures were canceled by the surgeon (*n* = 85) and the anesthetist (*n* = 73). Although the difference did not reach statistical significance (Kruskal-Wallis H, *p* = 0.054), surgical indications were associated with a higher median waiting time (125 days) compared to anesthesia-related reasons (98 days) and protocol non-adherence (60 days) (Figure 5).

When surgeons postponed procedures, the leading reasons included the absence of a precise surgical indication (other reasons were: other recent surgical procedure, parent withdrawal of consent, skin inflammation, technical problems with the instruments) (Vittori et al., 2025). In contrast, cancelations by anesthesiologists were predominantly driven by respiratory and infectious conditions.

The main categories of canceled surgical procedures included the Andrological team (*n* = 74), which represented the largest category, followed by the General and Thoracic team (*n* = 46) and the Plastic and Maxillofacial team (*n* = 30). Fewer standard procedures involved the following teams: Dentistry (*n* = 13) and Urology (*n* = 13). Seven unique categories were identified to describe the canceled surgical procedures, indicating that the interventions were primarily concentrated in specific surgical domains, such as andrology and general surgery.

Regarding the type of day hospital, the majority of cases fell under “Day Hospital with multiple accesses” (*n* = 83). This was followed by “Day Hospital with single access” (*n* = 22).

Concerning differences between performed procedures and canceled procedures, we found that not-operated patients were younger than the operated patients (Kolmogorov–Smirnov test *p*-value <0.001, Mann-Whittney *p*-value: 0.025, Age median for not-operated patients: 6.54, Age median for operated patients: 7.26), differed for surgical sector (Chi-square test *p*-value: *p* < 0.001) and type of surgery (Chi-square test *p*-value: *p* < 0.001) but not for the hospitalization setting in which the surgery took place (Chi-square test *p*-value: 0.45).

### 3.1 Cluster analysis

We performed the cluster analysis by implementing the variables age, HospitalizationRegime, setcat, codcat, and the column “performed.” The FAMD technique (n_components = 2, n_iter = 7, copy = True, check_input = True, engine = ‘sklearn’, random_state = 42) extracted two principal components, which together explained 54.5 and 45.5% of the total variance, respectively.

For cluster tendency assessment, the Hopkins score yielded a value of 0.003, confirming that the data structure was appropriate for this analysis.

The results of the metrics used for determining the optimal number of clusters (*k*) are summarized in Table 1.

**Table 1:** Clusters metrics.

*k*	Silhouette score	Davies-Bouldin score	Calinski-Harabasz score
2	0.0520	0.946	2,848.191
3	0.696	0.447	10,119.546
4	0.643	0.493	8,509.300
5	0.675	0.423	14,006.806
6	0.694	0.403	15,392.167
7	0.706	0.344	19,178.935
8	0.727	0.396	27,668.373
9	0.675	0.486	26,398.428
10	0.679	0.508	28,082.092

**Figure 6:**
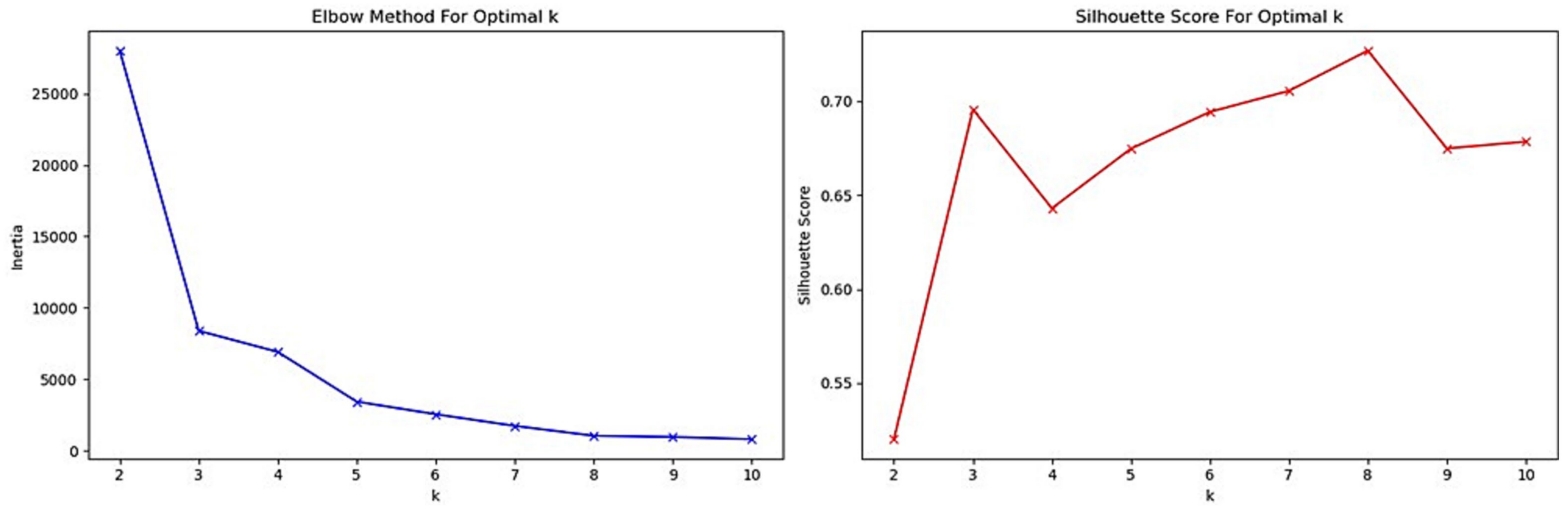
Elbow method and silhouette score for optimal *k*.

Based on the elbow method, a combination of these metrics was used to facilitate the subsequent statistical analysis, and *k* = 3 was selected as the optimal number of clusters. Results from the Elbow method and silhouette score are shown in Figure 6.

**Figure 7:**
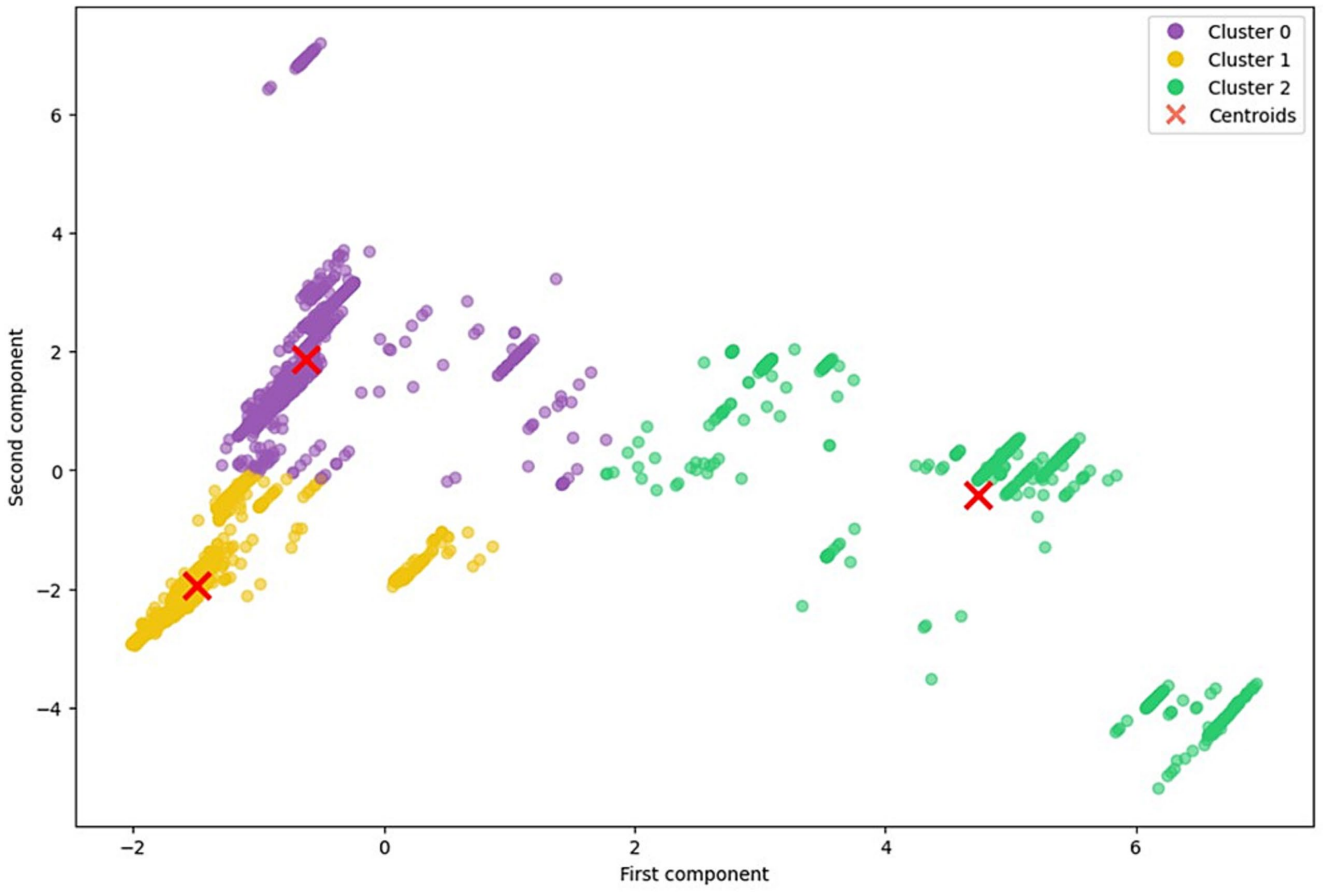
Scatter plots of FAMD components and clusters.

Figure 7 illustrates the three selected clusters based on the FAMD-derived components. The plot shows a separation among clusters in the reduced feature space, with red crosses indicating the corresponding cluster centroids.

### 3.2 Explainable AI approach

To better explain the results of the cluster analysis, we employed an explainable clustering approach by training a Random Forest classifier on the cluster’s membership classes and then calculating each feature’s importance.

**Figure 8:**
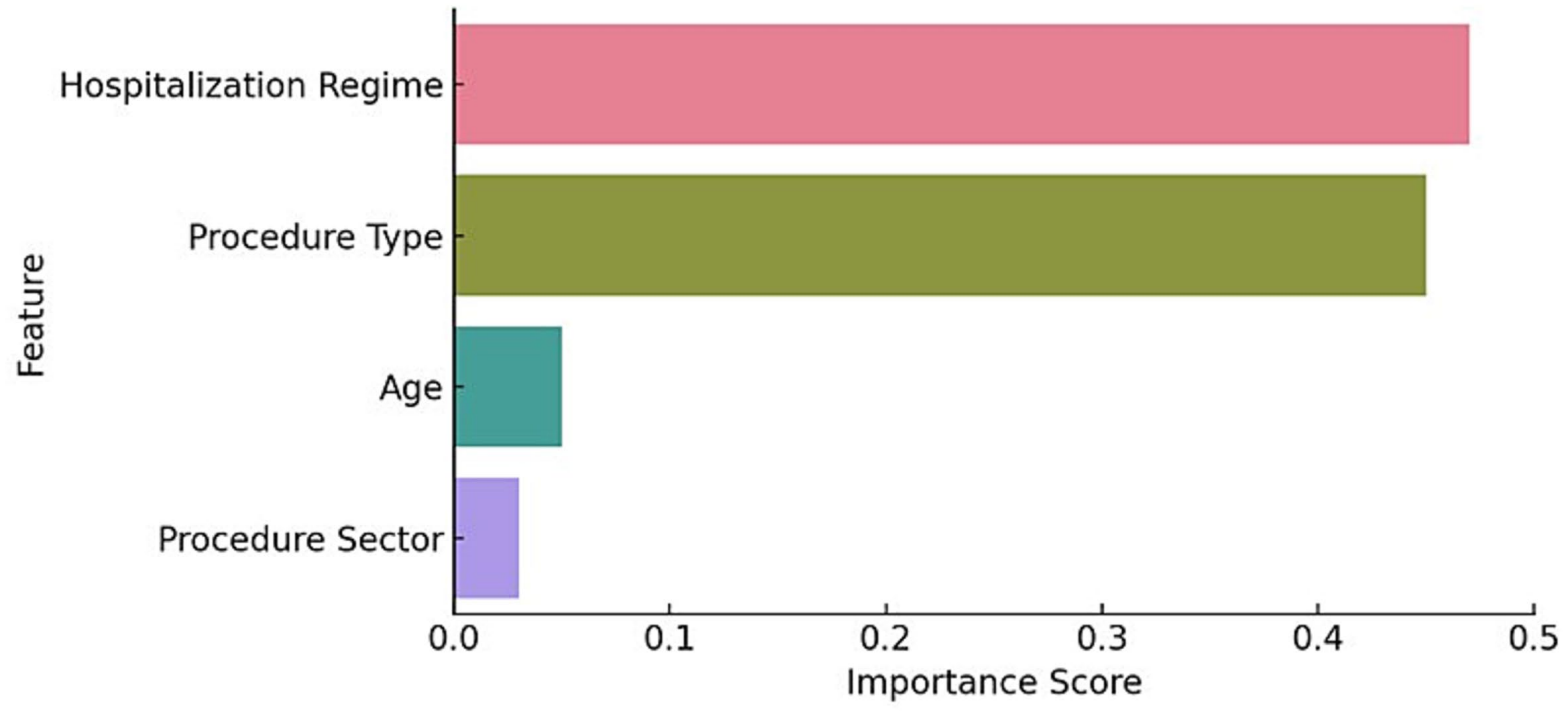
Random forest’s feature importance plot.

Figure 8 illustrates Random Forest’s feature importance plot. Hospitalization Regime (0.47) and Procedure Type (0.45) were the most influential variables in cluster assignment, whereas age and procedure sector showed a substantially smaller contribution.

## 4 Discussion

In our analysis, we found that the last-minute case cancelation rates were approximately 4%. This is in contrast to Ben Mansour et al. (2023), who found a rate of 9% or less, which is lower than the accepted average rate of 5% (Kaddoum et al., 2016). This data is even more significant considering that part of the dataset refers to the height of the COVID-19 pandemic, without there having been a dramatic increase in last-minute cancelations of procedures (Vittori et al., 2025; Vittori et al., 2020). One in five cases was canceled due to respiratory problems. Furthermore, protocol issues were 2% or less than in other analyses (Ben Mansour et al., 2023). An intriguing finding in our case series is the high percentage of incorrect indications for surgery. This highlights the need for a thorough reassessment of the clinical and diagnostic pathway leading up to the surgical intervention. These results highlight distinct clinical, administrative, and procedural considerations that influence cancelation decisions across professional roles involved in surgical patient management.

The clustering analysis aimed to identify distinct groups of patients based on surgical characteristics and outcomes. Specifically, we identified three distinct clusters of patients, revealing unique patterns in surgical teams and pathways. Cluster 0 predominantly comprised patients undergoing procedures performed by the following teams: Andrology, General and Thoracic Surgery, and Plastic and Maxillofacial Surgery. This cluster also had a significant proportion of day hospital cases with multiple accesses, reflecting the complexity of the patients and their management needs in these domains. Patients requiring multiple accesses likely have comorbidities that place them at greater risk of last-minute cancelations. To better validate the model, we calculate the importance scores of the features to provide insights into the model’s decision-making process, identifying the most influential factors and enhancing the model’s predictive accuracy while reducing uncertainty. Feature importance analysis revealed that the type of path on which the procedure took place had a significantly higher impact on predicting the classes of the clusters.

These data are important for two main reasons. First, ambulatory and day-case surgery are increasingly adopted—particularly in pediatric care—because of their well-recognized advantages, including reduced family stress, lower rates of perioperative infections, and decreased healthcare costs (Lerman, 2019). Second, operating room utilization can be optimized, with target saturation rates approaching 85%. In this context, the use of predictive models to anticipate and minimize last-minute cancelations may help hospitals approach this benchmark more effectively. Furthermore, in some institutions—such as the Ospedale Pediatrico Bambino Gesù IRCCS—the facilities dedicated to ambulatory and day surgery are physically separated from the main hospital, adding organizational dimension to the management of surgical scheduling and cancelations. This organizational separation offers clear advantages in terms of cost containment and infection prevention. However, it does not provide a safeguard pathway for patients whose procedures are canceled at the last minute and who might require conversion to ordinary hospitalization. The analysis of this data through artificial intelligence can offer hospitals valuable insights to implement targeted strategies aimed at reducing the rate of last-minute cancelations. Potential interventions include ensuring continuity between the surgeon who evaluates the surgical indication and the one performing the procedure, implementing telemedicine services for preoperative follow-up and monitoring, and providing families with clear informational materials—also in multimedia formats—to improve preparation and adherence to preoperative instructions (Vittori et al., 2025; Cascella et al., 2023).

In addition, these findings represent the first Italian data on the application of machine learning to the analysis of last-minute cancelations in pediatric ambulatory and day-case surgery, extending beyond the investigation of risk factors for cancelations previously reported by Vittori et al. (2025). The only other pediatric study applying ML to investigate last-minute surgical cancelations reported markedly different findings. In that analysis, the main factors associated with cancelations included the impact of the COVID-19 pandemic, environmental variables such as average wind speed and rainfall, and issues related to the completion of pre-anesthetic assessments (Li et al., 2024).

In Italy, the topic of resource optimization is a particularly sensitive issue, given the nature of the National Health System, which provides free or nearly free assistance to all patients (La Torre and Federici, 2017). The increase in outpatient and day surgery procedures is one of the key strategies for reducing the costs of the National Health System, which risks becoming unsustainable (Vittori et al., 2023). Therefore, ML can serve as a valuable tool for the continuous improvement of care quality, enabling the real-time identification and correction of critical elements within the clinical and organizational decision-making process.

### 4.1 Limitations

The main limitation of our study is that it represents a secondary analysis of retrospectively collected data. Nevertheless, the use of electronic medical records allowed the collection of high-quality and comprehensive information. A second limitation is the monocentric design, although the study was conducted at the Ospedale Pediatrico Bambino Gesù IRCCS, the largest pediatric hospital in Europe. At the same time, this context represents both a strength and a potential limitation: a strength because of the availability of a large volume of high-quality clinical data, and a limitation because the institution operates under particularly rigorous quality and safety standards, which may influence organizational dynamics and limit the generalizability of the findings to other settings (Vatican News, 2026). Indeed, the high number of last-minute cancelations may, in part, reflect the hospital’s exceptionally high standards of professionalism, well-structured care pathways, and rigorous accreditation requirements (Vittori et al., 2023; Joint Commission International, 2026). This makes it necessary to use the data from this study as a starting point for improving the quality, first and foremost, of our hospital.

## 5 Conclusion

Day surgery and ambulatory procedures in pediatric anesthesia represent an effective strategy for optimizing the use of healthcare resources while maintaining high standards of safety and quality of care. In this perspective, the use of ML can provide valuable guidance. Patients requiring multiple preoperative visits, often reflecting greater comorbidity and the need for several specialist consultations, as well as those experiencing prolonged waiting times between diagnosis and the scheduled procedure, appear to be at the highest risk of last-minute cancelations. Integrating ML into electronic medical record systems and surgical waiting-list management platforms could therefore enable the generation of targeted alerts, helping clinicians identify high-risk patients and direct them toward protected and more closely monitored care pathways. In addition, certain surgical teams appear to have a higher risk of last-minute procedure cancelations, likely because they manage patients with greater clinical complexity and higher comorbidity burdens. Increased attention to diagnostic and therapeutic pathways, reduction of waiting times between diagnosis and surgery, and greater standardization of preoperative assessment processes may help reduce the incidence of last-minute cancelations.

## Data Availability

The datasets presented in this study can be found in online repositories. The names of the repository/repositories and accession number(s) can be found at: https://zenodo.org/records/13961157.

## References

[ref1] AGENAS (2024). L’Attività di Day Surgery nelle Regioni. Available online at: https://www.agenas.gov.it/i-quaderni-di-monitor-%E2%80%93-supplementi-alla-rivista/369-attivita-day-surgery (Accessed November 9, 2024).

[ref2] Aguilar E. J. Barbosa V. C. (2023). Shape complexity in cluster analysis. PLoS One 18:e0286312. doi: 10.1371/journal.pone.0286312, 37235568 PMC10218739

[ref3] Al Talalwah N. McIltrot K. H. (2019). Cancellation of surgeries: integrative review. J. Perianesth. Nurs. 34, 86–96. doi: 10.1016/j.jopan.2017.09.012, 29678319

[ref4] ASAHQ. (2024). Statement on ambulatory anesthesia and surgery. Available online at: https://www.asahq.org/standards-and-practice-parameters/statement-on-ambulatory-anesthesia-and-surgery (Accessed December 27, 2024).

[ref5] Bellini V. Cascella M. Cutugno F. Russo M. Lanza R. Compagnone C. . (2022). Understanding basic principles of artificial intelligence: a practical guide for intensivists. Acta. Biomed. 93:e2022297. doi: 10.23750/abm.v93i5.13626, 36300214 PMC9686179

[ref6] Ben Mansour M. Lassioued O. Chakroun S. Slimene A. Ben Youssef S. Ksiaa A. . (2023). Elective surgery cancelations in pediatric surgery: rate and reasons. BMC Pediatr. 23:383. doi: 10.1186/s12887-023-04184-x, 37528359 PMC10394773

[ref7] Caliński T. Harabasz J. (1974). A dendrite method for cluster analysis. Commun. Stat. 3, 1–27. doi: 10.1080/03610927408827101

[ref8] Cascella M. Scarpati G. Bignami E. G. Cuomo A. Vittori A. Di Gennaro P. . (2023). Utilizing an artificial intelligence framework (conditional generative adversarial network) to enhance telemedicine strategies for cancer pain management. J. Anesth. Analg. Crit. Care 3:19. doi: 10.1186/s44158-023-00104-8, 37386680 PMC10280947

[ref9] Compère V. Grognu A. Moriceau J. Dureuil B. (2017). Mobile phone text messaging reminder decreases the rate of nonattendance at a preoperative anaesthesia clinic. Eur. J. Anaesthesiol. 34, 566–567. doi: 10.1097/EJA.0000000000000607, 28682817

[ref10] Davies D. L. Bouldin D. W. (1979). A cluster separation measure. IEEE Trans. Pattern Anal. Mach. Intell. 1, 224–227. doi: 10.1109/TPAMI.1979.4766909 21868852

[ref11] Ezike H. Ajuzieogu V. Amucheazi A. (2011). Reasons for elective surgery cancellation in a referral hospital. Ann. Med. Health Sci. Res. 1, 197–202. 23209975 PMC3507108

[ref12] Ferschl M. B. Tung A. Sweitzer B. Huo D. Glick D. B. (2005). Preoperative clinic visits reduce operating room cancellations and delays. Anesthesiology 103, 855–859. doi: 10.1097/00000542-200510000-00025, 16192779

[ref13] Joint Commission International. (2026). Find accredited international organizations. Available online at: https://www.jointcommission.org/en/about-us/recognizing-excellence/find-accredited-international-organizations?rfkid_7:content_filters=country:eq:Italy;acc_org_accredited_programs_filter:eq:Academic+Medical+Center+Hospital+Program (Accessed March 13, 2026).

[ref14] Kaddoum R. Fadlallah R. Hitti E. El-Jardali F. El Eid G. (2016). Causes of cancellations on the day of surgery at a tertiary teaching hospital. BMC Health Serv. Res. 16:259. doi: 10.1186/s12913-016-1475-6, 27412041 PMC4944432

[ref15] Koh W. X. Phelan R. Hopman W. M. Engen D. (2021). Cancellation of elective surgery: rates, reasons and effect on patient satisfaction. Can. J. Surg. 64, E155–E161. doi: 10.1503/cjs.008119, 33666393 PMC8064262

[ref16] Koushan M. Wood L. C. Greatbanks R. (2021). Evaluating factors associated with the cancellation and delay of elective surgical procedures: a systematic review. Int. J. Qual. Health Care 33:mzab092. doi: 10.1093/intqhc/mzab092, 34100548

[ref17] Kruskal W. H. Wallis W. A. (1952). Use of ranks in one-criterion variance analysis. J. Am. Stat. Assoc. 47, 583–621. doi: 10.2307/2280779

[ref18] La Torre G. Federici A. (2017). How to not detonate the bomb: the case of the Italian National Health Service. Public Health 153, 178–180. doi: 10.1016/j.puhe.2017.09.002 29050810

[ref19] Lerman J. (2019). Pediatric ambulatory anesthesia: an update. Curr. Opin. Anaesthesiol. 32, 708–713. doi: 10.1097/ACO.0000000000000787 31503033

[ref20] Li C. Li Z. Huang S. Chen X. Zhang T. Zhu J. (2024). Machine learning-based approach to predict last-minute cancellation of Pediatric day surgeries. Comput. Inform. Nurs. 42, 363–368. doi: 10.1097/CIN.0000000000001110, 38453534

[ref21] Olson R. P. Dhakal I. B. (2015). Day of surgery cancellation rate after preoperative telephone nurse screening or comprehensive optimization visit. Perioper Med (Lond) 4:12. doi: 10.1186/s13741-015-0022-z, 26664719 PMC4674935

[ref22] Pagès J. (2014). Multiple Factor Analysis by Example Using R. New York: Chapman and Hall/CRC, 272.

[ref23] Pohlman G. D. Staulcup S. J. Masterson R. M. Vemulakonda V. M. (2012). Contributing factors for cancellations of outpatient pediatric urology procedures: single center experience. J. Urol. 188, 1634–1638. doi: 10.1016/j.juro.2012.03.111 22910272

[ref24] Pollard J. B. Olson L. (1999). Early outpatient preoperative anesthesia assessment: does it help to reduce operating room cancellations? Anesth. Analg. 89, 502–505. doi: 10.1097/00000539-199908000-00048, 10439775

[ref25] RPubs. (2025). Document. Available online at: https://rpubs.com/aliro/831890 (Accessed July 10, 2025).

[ref26] Solak A. K. Pandza H. Beciragic E. Husic A. Tursunovic I. Djozic H. (2019). Elective case cancellation on the day of surgery at a general Hospital in Sarajevo: causes and possible solutions. Mater. Sociomed. 31, 49–52. doi: 10.5455/msm.2019.31.49-52, 31213956 PMC6511384

[ref27] Vatican News. (2026). Vatican children’s hospital ranked 6th best in the world. Available online at: https://www.vaticannews.va/en/vatican-city/news/2026-02/bambino-gesu-children-hospital-ranking-best-world-newsweek.html (Accessed March 13, 2026).

[ref28] Vittori A. Cascella M. (2024). Dataset_Surgical_Procedure_Children. Geneva: Zenodo.

[ref29] Vittori A. Iyer R. S. Cascella M. Tarquini R. Francia E. Mascilini I. . (2025). Last-minute cancellations in pediatric ambulatory and day surgeries in Italy: prevalence and risk factors. Paediatr. Anaesth. 35, 439–445. doi: 10.1111/pan.15093, 40084967 PMC12060080

[ref30] Vittori A. Lerman J. Cascella M. Gomez-Morad A. D. Marchetti G. Marinangeli F. . (2020). COVID-19 pandemic acute respiratory distress syndrome survivors: pain after the storm? Anesth. Analg. 131, 117–119. doi: 10.1213/ANE.0000000000004914, 32541584 PMC7199772

[ref31] Vittori A. Tritapepe L. Chiusolo F. Rossetti E. Cascella M. Petrucci E. . (2023). Unplanned admissions after day-case surgery in an Italian third-level pediatric hospital: a retrospective study. Perioper. Med. (Lond.) 12:53. doi: 10.1186/s13741-023-00342-y, 37752610 PMC10523757

[ref32] Wolfler A. De Silvestri A. Camporesi A. Ivani G. Vittori A. Zadra N. . (2020). Pediatric anesthesia practice in Italy: a multicenter national prospective observational study derived from the APRICOT trial. Minerva Anestesiol. 86, 295–303. doi: 10.23736/S0375-9393.19.14126-0, 31820874

